# Perovskite-Enhanced Silicon-Nanocrystal Optoelectronic Synaptic Devices for the Simulation of Biased and Correlated Random-Walk Learning

**DOI:** 10.34133/2020/7538450

**Published:** 2020-09-02

**Authors:** Yiyue Zhu, Wen Huang, Yifei He, Lei Yin, Yiqiang Zhang, Deren Yang, Xiaodong Pi

**Affiliations:** ^1^State Key Laboratory of Silicon Materials and School of Materials Science and Engineering, Zhejiang University, Hangzhou, Zhejiang 310027, China; ^2^School of Materials Science and Engineering, Henan Institute of Advanced Technology, Zhengzhou University, Zhengzhou, Henan 450001, China; ^3^Institute of Advanced Semiconductors, Hangzhou Innovation Center, Zhejiang University, Hangzhou, Zhejiang 311215, China

## Abstract

Silicon- (Si-) based optoelectronic synaptic devices mimicking biological synaptic functionalities may be critical to the development of large-scale integrated optoelectronic artificial neural networks. As a type of important Si materials, Si nanocrystals (NCs) have been successfully employed to fabricate optoelectronic synaptic devices. In this work, organometal halide perovskite with excellent optical asborption is employed to improve the performance of optically stimulated Si-NC-based optoelectronic synaptic devices. The improvement is evidenced by the increased optical sensitivity and decreased electrical energy consumption of the devices. It is found that the current simulation of biological synaptic plasticity is essentially enabled by photogating, which is based on the heterojuction between Si NCs and organometal halide perovskite. By using the synaptic plasticity, we have simulated the well-known biased and correlated random-walk (BCRW) learning.

## 1. Introduction

Computers have gained worldwide popularity over the past few decades. The von Neumann architecture on which computers are based, however, has been increasingly limiting the further development of computers [1–4]. Nowadays, an artificial neural network (ANN) is regarded as one of the most important alternative architectures for computers in the future since neuromorphic computing based on the ANN is capable of parallelly processing information and executing brain-like operations such as learning and memorizing with ultralow energy consumption [5–13]. For the construction of an ANN, artificial synapses (i.e., synaptic devices) are critical given the fact that information transmitted among neurons basically relies on synapses in a biological neural system [14, 15]. Until now, different kinds of synaptic devices have been proposed by using various functional materials such as metal oxide films [16–23], organic films [24, 25], two-dimensional layered films [26–30], and semiconductor nanostructures [31–35]. It is noteworthy that silicon nanocrystals (Si NCs) have been successfully employed in the fabrication of synaptic devices as a type of important Si materials [36–39]. They exemplified the great promise for the development of Si-based optoelectronically integrated ANNs, which would facilitate widely deployable neuromorphic computing.

An optoelectronic synaptic device is usually stimulated by presynaptic optical spikes [36]. Under a bias, the device exhibits optical-spike-induced postsynaptic current, consuming electrical energy. High optical sensitivity and low electrical energy consumption are highly demanded by an optoelectronic synaptic device [37, 40, 41]. Hence, significant efforts for rendering Si-NC-based optoelectronic synaptic devices better optical sensitivity and lower electrical energy consumption are well justified. It is known that organometal halide perovskite is emerging as an excellent optoelectronic material [15, 42–46]. A pronounced feature of the optical properties of organometal halide perovskite is its extremely strong optical absorption [47]. This inspires the route of hybridizing Si NCs with organometal halide perovskite to enhancing the optical sensitivity of Si-NC-based optoelectronic synaptic devices. The electronic coupling between organometal halide perovskite and Si NCs also likely helps to reduce the electrical energy consumption of Si-NC-based optoelectronic synaptic devices.

In this work, an organometal halide perovskite film is coated on a Si NC film, which is the channel of an optoelectronic synaptic transistor. It turns out that the organometal halide perovskite film not only significantly improves the optical sensitivity of the device but also reduces the electrical energy consumption of the device. Synaptic functionalities, for instance excitatory postsynaptic current (EPSC), paired-pulse facilitation (PPF), spike-number-dependent plasticity (SNDP), and spike-rate-dependent plasticity (SRDP) [48], can be all mimicked by using synaptic transistors which are based on the hybrid structure of organometal halide perovskite and Si NCs. Moreover, it is demonstrated that biased and correlated random-walk (BCRW) learning can be simulated by our synaptic devices.

## 2. Results and Discussion

**Figure 1:**
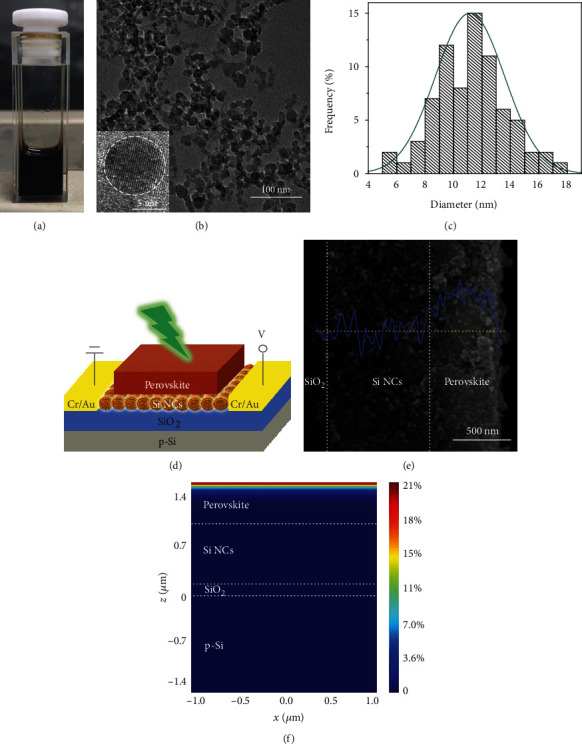
Characterization of Si NCs and a perovskite-enhanced Si NC synaptic device. (a) Photograph of B-doped Si NCs in ethanol. (b) Low-resolution TEM image of Si NCs. The inset is the high-resolution TEM image of a Si NC. (c) Size distribution with a Gaussian fitting for Si NCs. The mean size of Si NCs is ~11.2 nm. (d) Schematic of a perovskite-enhanced Si NC synaptic device. The light that works as the optical stimulation incidents from the top of the device. (e) Cross-section SEM image of the synaptic device and EDS line scanning of the element of Pb. The thickness of the perovskite film and Si NC film is ~500 nm and~850 nm, respectively. (f) Cross-section distribution of the ratio of absorbed power per unit volume to total incident power of the synaptic device under the illumination of 532 nm laser.

Figure 1(a) shows the photograph of a Si NC solution, in which Si NCs are dispersed in ethanol to render a concentration of ~10 mg/ml. The good dispersion of Si NCs is enabled by their heavy B doping [49]. The low-resolution transmission electron microscopy (TEM) image of Si NCs is shown in Figure 1(b) together with the high-resolution TEM image of an individual Si NC as the inset. Statistical analysis on the size of Si NCs indicates a mean size of ~11.2 nm and a standard deviation of ~2.4 nm (Figure 1(c)).

Optoelectronic synaptic devices with the structure schematically illustrated in Figure 1(d) are fabricated by employing the above-mentioned Si NCs. The Si NC solution is first spin-coated on SiO_2_/Si with Cr/Au electrodes. The resulting Si NC film in the area (10 *μ*m long and 120 *μ*m wide) between Cr/Au electrodes acts as the conducting material of an optoelectronic synaptic device. A film of the perovskite of MAPbI_3_ is then formed on the top of the Si NC film. From the cross-section scanning electron microscopy (SEM) image (Figure 1(e)), it is found that the perovskite film and Si NC film are ~500 nm and ~850 nm thick, respectively. The bilayer structure may be further clarified by energy dispersive spectrometer (EDS) line scanning for the element of Pb. A finite difference time domain (FDTD) method is used to analyze the optical absorption of the device for light with the wavelength of 532 nm incident on the top (i.e., perovskite). The cross-section distribution of the ratio of absorbed power per unit volume to total incident power of the synaptic device is shown in Figure 1(f). The perovskite film is found to be responsible for nearly 100% of the absorbed light. This is consistent with the fact that the absorption coefficient of a perovskite film is very large (~105 cm^−1^ at 532 nm) [47].

**Figure 2:**
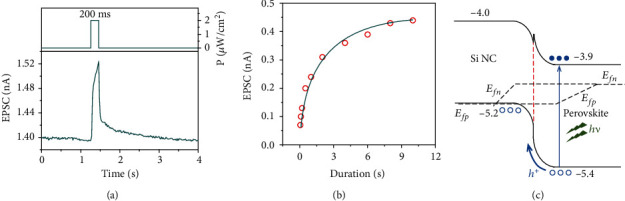
EPSC and working principle of the synaptic device. (a) EPSC stimulated by a 532 nm laser spike with the duration of 200 ms and the power density of 2 *μ*W/cm^2^. (b) Dependence of EPSC on the duration of optical laser spike. (c) Schematic of the band alignment between Si NCs and perovskite.

Figure 2(a) shows the EPSC of a synaptic device at 3 V induced by an optical spike with the wavelength of 532 nm, the duration of 200 ms, and the power density of 2 *μ*W/cm^2^. The maximum value of EPSC (~0.12 nA) is reached just before the optical spike stops. The decay time (which is defined as the time required to reduce the EPSC from 90% to 10% of the original value) is measured to be ~0.4 s [50]. It is found that the EPSC increases as the duration of the optical spike increases, which is shown in Figure 2(b). The saturation of the EPSC occurs when the duration is larger than ~10 s. Such a behavior of the EPSC for the synaptic device is similar to that for a biological synapse [51]. Figure S1 shows the EPSC of twelve devices. The standard deviation of the EPSC is ~5.7% of the average EPSC, which is better than that reported by Yang et al. [52]. This indicates the good repeatability of our devices.

Figure 2(c) schematically illustrates the band alignment of the heterojunction formed between perovskite and Si NCs. Please note that Si NCs are *p*-type degenerated semiconductors [38, 53], while the perovskite of MAPbI_3_ is a *n*-type semiconductor [54, 55]. When an optical spike is incident on the top of the heterojunction, photo-generated holes and electrons appear in perovskite. The built-in electric field of the heterojunction allows photo-generated holes to move into Si NCs, while leaving photo-generated electrons in perovskite. As a result, the hole concentration in the Si NC film increases, giving rise to the EPSC. Since photo-generated holes transferred into Si NCs are physically separated from photo-generated electrons in perovskite, they have longer lifetime. Hence, we observe the slow decay of the EPSC. This is the so-called the photogating effect [55, 56].

In order to further clarify the working mechanism, we have fabricated a transistor only based on the Si NC film and a transistor only based on the perovskite film, as shown in Figure S2. It is found that the conductivity of the Si NC film is quite comparable to that of the perovskite film. Liu et al. [57] showed that for two layers with similar conductivity, the layer near the insulator plays a decisive role in the charge transport of a transistor. Therefore, the Si NC layer should be regarded as the conductive channel of a synaptic device in the current work. We have also compared the photocurrent of a Si NC synaptic device with that of a perovskite-enhanced Si NC synaptic device. The Si NC synaptic device has a negative photocurrent [37], while the perovskite-enhanced Si NC synaptic device has a large positive photocurrent. Such comparison indicates that under illumination, the carrier concentration in the perovskite-enhanced Si NC synaptic device is clearly larger than that of the Si NC synaptic device. Hence, it may be inferred that photo-generated holes in perovskite move to Si NCs, leading to the increase of the hole concentration of the Si NC conductive channel in the current work. This is in fact consistent with the band alignment of the heterojunction between perovskite and Si NCs (Figure 2(c)).

The sensitivity of an optoelectronic synaptic device to optical stimulation may be evaluated by responsivity (*R*), which is the ratio of the photocurrent (*I*_ph_) to the power of optical stimulation (*P*). In the current work, we obtain the highest responsivity of 18 A/W when the device is stimulated by an optical spike to exhibit the saturated EPSC. By comparing the current device (Figure S3) with a synaptic device only based on Si NCs under the illumination with the same power density, we find that the responsivity of the current device is more than three orders of magnitude larger than that of the synaptic device only based on Si NCs [37]. This indicates that perovskite with excellent optical absorption indeed enhances the sensitivity of a Si NC synaptic device. In addition, the electrical energy consumption per synaptic event for a synaptic device based on the hybrid structure of Si NCs and perovskite is calculated by using

(1) $$\begin{array}{c} \text{d}E=V\times I\times \text{d}t, \end{array}$$

in which *V* is the bias (3 V) and *I* is the current induced by a spike at the time of *t* [58]. It is found that the lowest electrical energy consumption of 0.9 nJ is obtained at the shortest duration of 200 ms in this work. As shown in Figure S4, a lower bias (e.g., 0.01 V) may be applied to the device to get even lower electrical energy consumption (~0.11 pJ), which is more than three orders of magnitude smaller than that of a Si NC synaptic device [37]. Therefore, we conclude that perovskite helps reduce the electrical energy consumption of a Si NC synaptic device.

**Figure 3:**
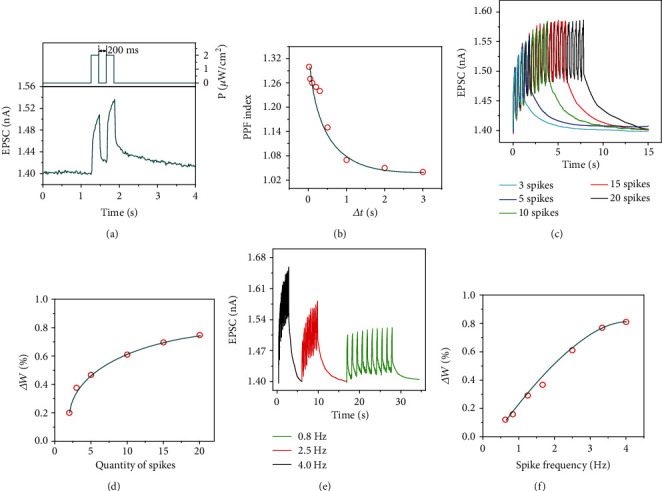
Synaptic functionalities of the synaptic device. (a) EPSC of the synaptic device stimulated by two sequential 532 nm laser spikes with a 200 ms Δ*t*. One spike duration is 200 ms, and the power density of the laser is 2 *μ*W/cm^2^. (b) Dependence of the PPF index on Δ*t*. (c) EPSC induced by 532 nm laser spikes with different spike quantities. The spike duration and Δ*t* are both 200 ms. (d) Dependence of the synaptic weight change (Δ*W*) on the quantity of spikes. (e) EPSC induced by ten successive 532 nm laser spikes with different spike frequencies. The spike duration is 200 ms. (f) Dependence of Δ*W* on the spike frequency.

Figure 3(a) shows the EPSC of a perovskite-enhanced Si NC synaptic device induced by two sequential optical spikes with a 200 ms interval time (Δ*t*). We can find that the EPSC induced by the second optical spike (*A*_2_) is bigger than that induced by the first one (*A*_1_). This is the so-called PPF, which is a characteristic manifestation of STP in a biological neural system. If we define the PPF index as the ratio of *A*_2_ and *A*_1_, we can work out the dependence of the PPF index on Δ*t* (Figure 3(b)). It is found that the PPF index decreases as Δ*t* increases. When Δ*t* is very small, the PPF index reaches about 130%. However, the PPF index tends to be 100% when Δ*t* exceeds ~3 s. This change is consistent with the learning process enabled by biological synapses [23].

The EPSC induced by optical spikes with different spike quantities is shown in Figure 3(c). The quantity of optical spikes ranges from 2 to 20. It is clear that the EPSC increases when the quantity of optical spikes increases. Since the synaptic weight change (Δ*W*) can be calculated by using

(2) $$\begin{array}{c} \Delta W=\left(A_{n}-A_{1}\right)/A_{1}, \end{array}$$

where *A*_*n*_ is the EPSC measured at the end of last optical spike. We may examine the dependence of Δ*W* on the quantity of spikes. It turns out that Δ*W* increases and then has a tendency to saturate at ~75% as the quantity of optical spikes increases (Figure 3(d)). Such a phenomenon may be called SNDP [48]. In addition, we find that SRDP also occurs to our perovskite-enhanced Si NC synaptic devices.  Figure 3(e) shows that the EPSC of a perovskite-enhanced Si NC synaptic device increases when the optical spike frequency increases from 0.8 to 4.0 Hz. Δ*W* also increases and then saturates at ~81% as the spike frequency increases (Figure 3(f)).

**Figure 4:**
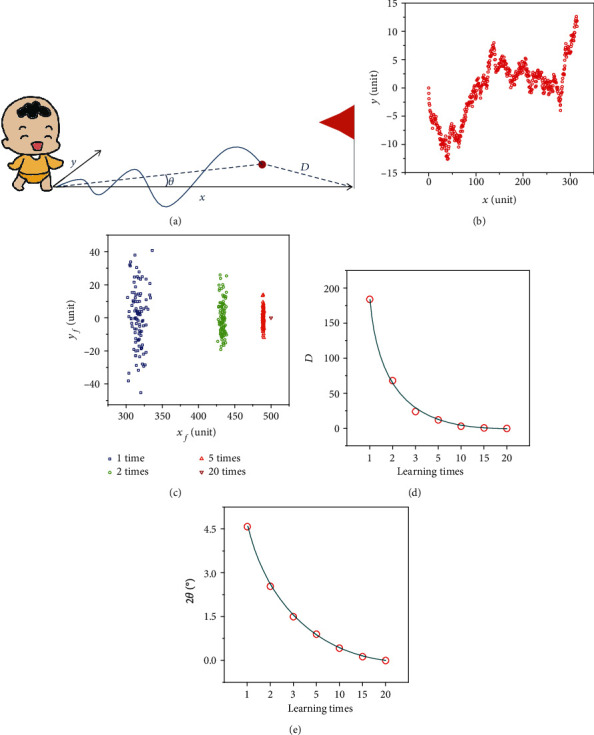
Simulation of BCRW. (a) Schematic of a BCRW. The direction of each forward step ranges from -90° to 90°. The coordinate of each step is denoted by (*x*, *y*). The distance between the final point and the target is marked as *D*. The angel between the line of the starting point to the final point and that of the starting point to the target is *θ*. (b) Footprints of the toddler's 500-step walk for *n* = 1. (c) The end points of 100 experiments of BCRWs after different learning times. (d) Dependence of *D* on the learning times. (e) Dependence of 2*θ* on the learning times.

Now we move to demonstrate that our perovskite-enhanced Si NC synaptic devices may be used to simulate practical learning. It is well known that biased and correlated random-walk (BCRW) learning is usually carried out by animals and humans [59–61].  Figure 4(a) shows an example of BCRW learning, in which a toddler learns to walk toward a target. The target is 500 units away from the starting point of the toddler, who walks one unit per step. The direction of each newly started step is evaluated by its angle (*α*) with respect to the horizontal line between the starting point and the target. *α* may randomly change from -90° to 90°. Since the range of choice of the absolute value of *α* (∣*α*∣) is closer to zero for better training in the BCRW learning, ∣*α*∣ may be correlated to learning times (*n*). *n* can be readily denoted by the quantity of optical spikes for a perovskite-enhanced Si NC synaptic device. Hence, we can write down

(3) $$\begin{array}{c} \left|\alpha \right|=180^{{}^{\circ}}/\left(1+S\left(n\right)\right)-90^{{}^{\circ}}, \end{array}$$

where *S*(*n*) is given by the normalization of Δ*W*(*n*) with respect to its saturated value (i.e., Δ*W*(20)). We assume that the toddler totally walks 500 steps, reaching a final point denoted by (*x*_*f*_, *y*_*f*_). The values of *x*_*f*_ and *y*_*f*_ are obtained by using

(4) $$\begin{array}{c} x_{f}=\underset{500}{\sum }\cos\left(\text{randint}\left(90^{{}^{\circ}}-\frac{180^{{}^{\circ}}}{1+S\left(n\right)},\frac{180^{{}^{\circ}}}{1+S\left(n\right)}-90^{{}^{\circ}}\right)\right),\\ y_{f}=\underset{500}{\sum }\sin\left(\text{randint}\left(90^{{}^{\circ}}-\frac{180^{{}^{\circ}}}{1+S\left(n\right)},\frac{180^{{}^{\circ}}}{1+S\left(n\right)}-90^{{}^{\circ}}\right)\right), \end{array}$$

where randint(*a*, *b*) randomly chooses a number between *a* and *b*.  Figure 4(b) representatively shows the footprints of the toddler's 500-step walk for *n* = 1. The final point the toddler reaches is (314, 12). We define the distance between the final point and the target as *D*. The angel between the line of the starting point to the final point and that of the starting point to the target is *θ*. *D* and 2*θ* may be well used to evaluate the deviation of the final point with respect to the target.

Figure 4(c) shows the statistical results on the final points the toddler reaches after 1, 2, 5, or 20 times learning. It is clear that with the increase of training times, *x*_*f*_ and y_*f*_ more closely approach 500 and zero, respectively. The dependence of *D* and 2*θ* on the learning times is shown in Figures 4(d) and 4(e), respectively. When the learning times increases from 1 to 20, the average *D* (2*θ*) of 100 experiments of BCRWs decreases from 184 units (2.3°) to 0 (0). Such results mean that after more training, the toddler is able to walk to the target with less deviation, consistent with the biological model of BCRW learning [61]. Please note that the SNDP rather than a single perovskite-enhanced Si NC synaptic device is used to simulate the BCRW learning here. In order to take the full advantage of the SNDP through hardware implementation, perovskite-enhanced Si NC synaptic devices need to be interconnected with appropriate neuronal devices to form an ANN.

## 3. Conclusions

In summary, we have incorporated perovskite into Si-NC-based optoelectronic synaptic devices. Synaptic functionalities such as EPSC, PPF, SNDP, and SRDP are simulated by these devices. It is found that perovskite clearly enhances the sensitivity of Si-NC-based synaptic devices to optical stimulation. Perovskite also lowers the electrical energy consumption of Si-NC-based synaptic devices. Significantly, we demonstrate that BCRW learning can be simulated by using the synaptic plasticity of perovskite-enhanced Si NC synaptic devices. The current work should inspire the development of novel optoelectronic devices based on the synergy of the traditional semiconductor of Si and the emerging semiconductors such as organometal halide perovskite.

## 4. Experimental Section

### 4.1. Material Preparation

Si NCs doped with boron at the nominal concentration of ~40% were synthesized by nonthermal plasma. The flow rates of SiH_4_, B_2_H_6_, and Ar introduced into the nonthermal plasma chamber and the plasma pressure were controlled to obtain the desired size and dopant concentration for Si NCs. The nonthermal plasma was generated with the power of 140 W and the frequency of 13.56 MHz [53]. The synthesized Si NCs were then dispersed in ethanol forming a 10 mg/ml solution. Before the use of the Si NC solution, a three-minute ultrasonication with a cell pulverizer at the power of 90 W was carried out. CH_3_NH_3_I and PbI_2_ were mixed with the molar ratio of 1 : 1 and dissolved in a mixed solution of N,N-dimethylformamide (AR, 99.8%) and dimethyl sulfoxide (>99.9%) with the volume ratio of 4 : 1 [48].

### 4.2. Device Fabrication

We first deposited Cr/Au (10 nm/150 nm) electrodes on a silicon wafer with a 150 nm thick thermally grown SiO_2_ layer. The length and the width of the channel between the electrodes were 10 and 120 *μ*m, respectively. The Si NC solution was then spin-coated on the silicon wafer at 1500 rpm for 60 s. The spin-coating was repeated a few times. After each spin-coating, the resulting Si NC film was annealed at 100°C for 5 minutes [36]. The perovskite layer was spin-coated on the Si NC film at 4000 rpm for 35 s. Antisolvent chlorobenzene (>99.9%) was drop-coated during the spin-coating of the perovskite layer. Finally, an annealing process of 100°C for 15 minutes was taken [48].

### 4.3. Characterization

A JEM 2100F transmission electron microscope with an acceleration voltage of 200 kV was used to obtain TEM images. A Zeiss GeminiSEM 500 scanning electron microscope was used to obtain the SEM images and EDS line scanning. A semiconductor parameter analyzer (FS480, PDA Co. Ltd.) was used to measure the optoelectronic synaptic devices. A Rigol DG5100 arbitrary function generator was used to modulate TTL-controlled optical shutters, rendering a series of optical spikes. All devices were measured after similar exposure to air. The FDTD simulation was performed by using the software of Lumerical Solutions Inc.

## Supplementary Materials

Supplementary Materials Figure S1: EPSC of twelve devices stimulated by a 532 nm laser spike with the duration of 200 ms and the power density of 2 *μ*W/cm^2^. Figure S2: (a) Schematic of a Si NC or perovskite transistor. (b) Transfer curve of a Si-NC or perovskite transistor at the drain voltage of 3 V. Figure S3: EPSC of a perovskite-enhanced Si NC synaptic device stimulated by a 532 nm laser spike with the duration of 200 ms and the power density of 1.3 mW/cm^2^ at the bias of 3 V. Figure S4: EPSC of a perovskite-enhanced Si NC synaptic device stimulated by a 532 nm laser spike with the duration of 200 ms and the power density of 2 *μ*W/cm^2^ at the bias of 0.01 V.
