# Supplementary material for: Perovskite-Enhanced Silicon-Nanocrystal Optoelectronic Synaptic Devices for the Simulation of Biased and Correlated Random-Walk Learning
Source: Research (Wash D C). 2020 Sep 2;2020:7538450. doi: 10.34133/2020/7538450 (PMC7510342; doi:10.34133/2020/7538450)
Supplement: Supplementary Materials — Figure S1: EPSC of twelve devices stimulated by a 532 nm laser spike with the duration of 200 ms and the power density of 2 μW/cm2. Figure S2: (a) Schematic of a Si NC or perovskite transistor. (b) Transfer curve of a Si-NC or perovskite transistor at the drain voltage of 3 V. Figure S3: EPSC of a perovskite-enhanced Si NC synaptic device stimulated by a 532 nm laser spike with the duration of 200 ms and the power density of 1.3 mW/cm2 at the bias of 3 V. Figure S4: EPSC of a perovskite-enhanced Si NC synaptic device stimulated by a 532 nm laser spike with the duration of 200 ms and the power density of 2 μW/cm2 at the bias of 0.01 V. [file 7538450.f1.docx]

**Supporting Information**

**Perovskite-Enhanced Silicon-Nanocrystal Optoelectronic Synaptic Devices for the Simulation of Biased and Correlated Random-Walk Learning**

Yiyue Zhu,^1^ Wen Huang,^1^ Yifei He,^1^ Lei Yin,^1^ Yiqiang Zhang,^2^ Deren Yang^1^ and Xiaodong Pi^1,3*^

*^1^State Key Laboratory of Silicon Materials and School of Materials Science and Engineering, Zhejiang University, Hangzhou, Zhejiang 310027, China*

*^2^School of Materials Science and Engineering, Henan Institute of Advanced Technology, Zhengzhou University, Zhengzhou, Henan 450001, China*

*^3^Institute of Advanced Semiconductors, ZJU-Hangzhou Global Scientific and Technological Innovation Center, Hangzhou, Zhejiang 311215, China*

^*^Corresponding author: Xiaodong Pi (email: xdpi@zju.edu.cn)





**Figure S1** EPSC of twelve devices stimulated by a 532 nm laser spike with the duration of 200 ms and the power density of 2 μW/cm^2^.


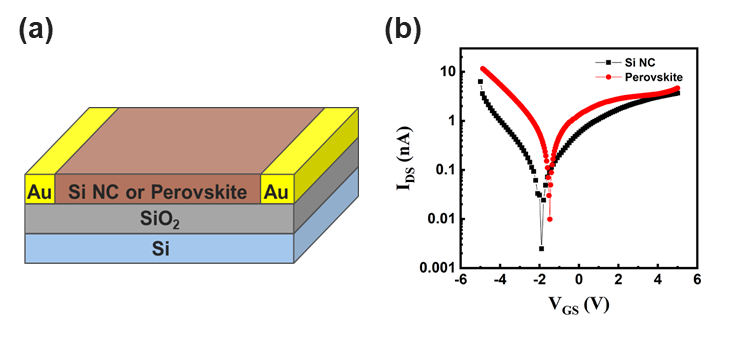


**Figure S2** (a) Schematic of a Si-NC or perovskite transistor. (b) Transfer curve of a Si-NC or perovskite transistor at the drain voltage of 3 V.


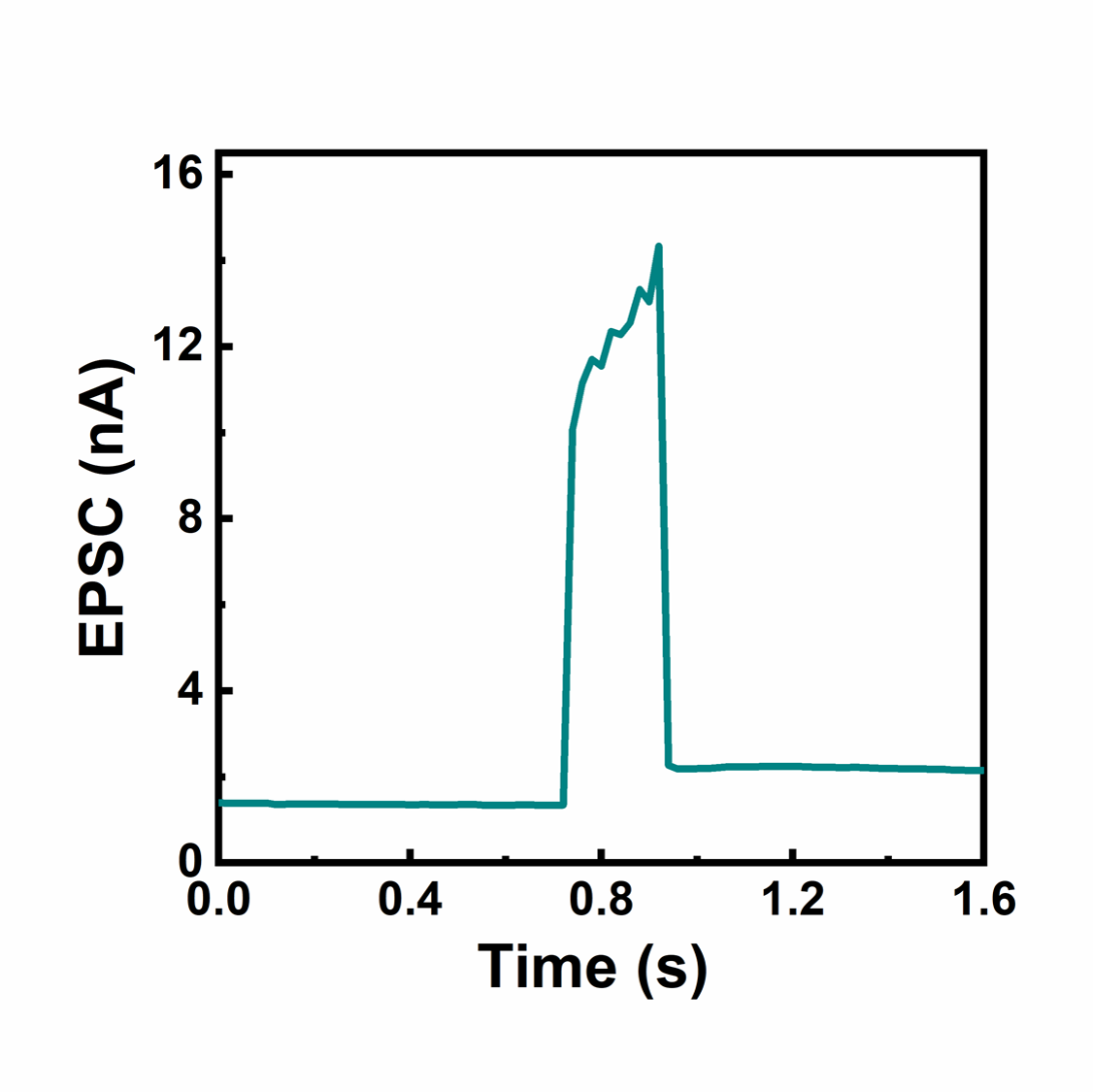


**Figure S3** EPSC of a perovskite-enhanced Si-NC synaptic device stimulated by a 532 nm laser spike with the duration of 200 ms and the power density of 1.3 mW/cm^2^ at the bias of 3 V.


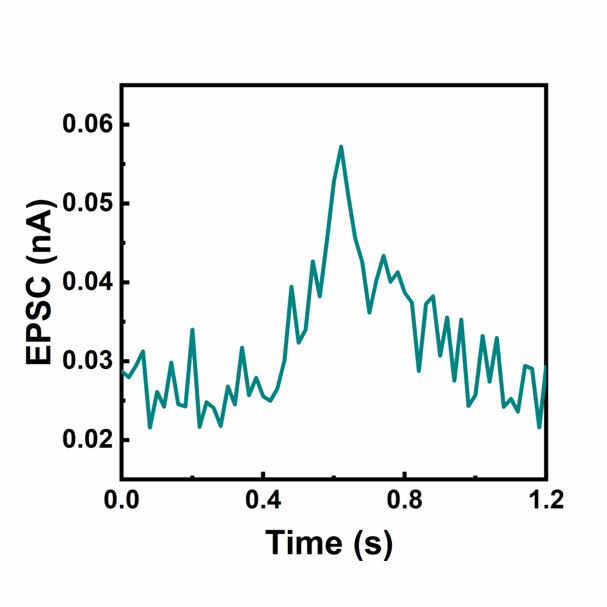


**Figure S4** EPSC of a perovskite-enhanced Si-NC synaptic device stimulated by a 532 nm laser spike with the duration of 200 ms and the power density of 2 μW/cm^2^ at the bias of 0.01 V.
